# Diagnostic pitfalls for the hand surgeon

**DOI:** 10.1186/1753-6561-9-S3-A3

**Published:** 2015-05-19

**Authors:** Lee Moon Keen

**Affiliations:** 1 Pantai Medical Centre, Kuala Lumpur, 59100, Malaysia

## 

Neurological and other medical disorders are occasionally mistaken for hand conditions. A correct diagnosis is crucial in planning and instituting treatment. This paper will discuss specific conditions and highlight some common pitfalls.

Neurological conditions like Parkinson’s Disease (PD) and stroke may present with signs and symptoms confined to the upper limb. Sensory complaints such as pain may predominate in the early stages of PD, amyotrophic lateral sclerosis and syringomyelia. Certain medical diseases may cause, predispose to or mimic entrapment syndromes. Connective tissue disorders and other immune-mediated neuropathies are ill-defined in the early stages and may pose diagnostic challenges.

Precise diagnosis requires global assessment of the patient, combined with attention to gait, stance and coordination. Electrophysiological studies can provide further elucidation. When in doubt, prompt referral to the appropriate specialist is indicated.

Early recognition will prevent unnecessary detours to the goal of proper treatment. In some cases, a timely diagnosis may avert unnecessary surgery.

